# Evaluation of vascular endothelial growth factor A and leukemia inhibitory factor expressions at the time of implantation in diabetic rats following treatment with Metformin and Pioglitazone

**DOI:** 10.18502/ijrm.v13i9.7666

**Published:** 2020-09-20

**Authors:** Ronak Zarei, Roshanak Aboutorabi, Bahman Rashidi, Nahid Eskandari, Parvaneh Nikpour

**Affiliations:** ^1^Department of Anatomical Sciences, Faculty of Medicine, Isfahan University of Medical Sciences, Isfahan, Iran.; ^2^Department of Immunology, Faculty of Medicine, Isfahan University of Medical Sciences, Isfahan, Iran.; ^3^Department of Genetics and Molecular Biology, Faculty of Medicine, Isfahan University of Medical Sciences, Isfahan, Iran.; ^4^Child Growth and Development Research Center, Research Institute for Primordial Prevention of Non-Communicable Disease, Isfahan University of Medical Sciences, Isfahan, Iran.

**Keywords:** Embryo implantation, Leukemia inhibitory factor, Vascular endothelial growth factor A, Metformin, Pioglitazone, Rats

## Abstract

**Background:**

Implantation requires intimate crosstalk between the embryo and uterus for a successful establishment of pregnancy. Type 2 diabetes mellitus may lead to implantation failure. The effect of diabetes and its therapeutic drugs on implantation is still largely unclear.

**Objective:**

To assess the endometrial expression changes of vascular endothelial growth factor A (VEGFA) and leukemia inhibitory factor (LIF), at the time of implantation in diabetic rats following treatment with Metformin and Pioglitazone.

**Materials and Methods:**

Twenty-eight 6-8-wk-old Wistar female rats weighing 200-250 gr were divided into four groups (n = 7/each). Type 2 diabetes was induced and Metformin and Pioglitazone were applied for 4 wk. The expression of VEGFA and LIF was measured by real-time reverse transcription-polymerase chain reaction and Western blot.

**Results:**

The relative expression of VEGFA transcript was higher in the diabetic (p = 0.02) and Metformin-treated (p = 0.04) rats compared to the control group. Furthermore, the VEGFA transcript level significantly reduced in Pioglitazone-treated diabetic rats (p = 0.03). LIF expression was elevated in the Metformin- and the Pioglitazone-treated rats and reduced in the diabetic group in comparison with the control group. Compared to the diabetic rats, the expression of LIF was significantly elevated in the Metformin- (p = 0.01) and Pioglitazone-treated (p = 0.03) groups.

**Conclusion:**

The expressions of LIF and VEGFA were altered in diabetic rats during implantation which may be associated with diabetic-related infertility. Pioglitazone is able to restore the VEGFA and LIF expressions to their baseline levels more efficiently than Metformin.

## 1. Introduction

Type 2 diabetes mellitus (T2DM) is caused by the insulin resistance in tissues and cells (1). Although the onset of T2DM is thought to be in the older ages, this condition also affects younger adults in the present days (2). The prevalence of diabetes mellitus is almost equal among male and female patients in different age groups (3); however, T2DM affects younger females at their fertile ages (< 40 yr) more than males (2).

The high level of serum insulin in T2DM impairs ovarian function during embryo implantation and is considered as a known factor affecting fertility rate in normal pregnancies (4). However, the regulatory role of other molecules such as hormones, growth factors, transcription factors, cytokines, lipid metabolites, and developmental genes in the dynamic of the highly complex embryo implantation process in the context of diabetes is yet to be fully elucidated (5). Leukemia inhibitory factor (LIF) is a cytokine mainly produced by the endometrial gland and necessary in making the endometrial epithelium receptive to blastocyst attachment (6). The vascular endothelial growth factor A (VEGFA) is a key regulator of angiogenesis and vascular function in the endometrium whose contribution to the implantation process has been well-established (7). Aberrant expression of both LIF and VEGFA has been previously reported in the context of diabetes. Changes have been reported in LIF expression in the endometrium, myoblasts and osteoblasts (8, 9), and VEGFA has been associated with the development of retinopathy, neuropathy, nephropathy and cardiovascular diseases in diabetic conditions (10).

Pioglitazone and Metformin are the two common drugs generally recommended for diabetic patients (11). Although it seems that these two drugs have beneficial effects on improving the reproductive and metabolic outcomes for women with polycystic ovary syndrome (12), their precise molecular effects on endometrial receptivity needs to be clarified.

There have been studies assessing the expression of LIF and VEGFA as implantation modulators (7, 13, 14); however, the expression changes of these molecules in diabetic conditions and the effects of diabetes treatment drugs on the endometrial expression of these molecules have not been well-understood. Therefore, the current study aimed to assess the endometrial expression changes of LIF and VEGFA at the time of implantation in diabetic rats following the treatment with Metformin and Pioglitazone.

## 2. Materials and Methods

### Animal models 

This experimental study was conducted on 6-8-wk-old Wistar rats weighing 200-250 gr provided by the Pasteur Institute of Iran. The animals were kept in standard conditions: 20-22°C in a temperature-controlled room, with 40-70% humidity; they were exposed to 12-hr light/dark cycle with free access to standard water and food. They were housed in the central animal house laboratory of Isfahan University of Medical Sciences.

### Diabetes induction and verification tests

Type 2 diabetes was induced by a combination of Nicotinamide (NA, Sigma-Aldrich, Germany) and Streptozotocin (STZ, Sigma-Aldrich, Germany) at a dose of 200 mg/kg and 60 mg/kg of body weight, respectively, via two intraperitoneal injections over 15 min (15). To verify the diabetes induction, fasting blood sugar (FBS) was tested from the dorsal vein of the rats using a glucometer (HemoCue Glucose 201+, Sweden) three days after the injection. FBS > 250 mg/dl was considered as diabetic animal models (16).

### Study design and sample collection

**Figure 1:**
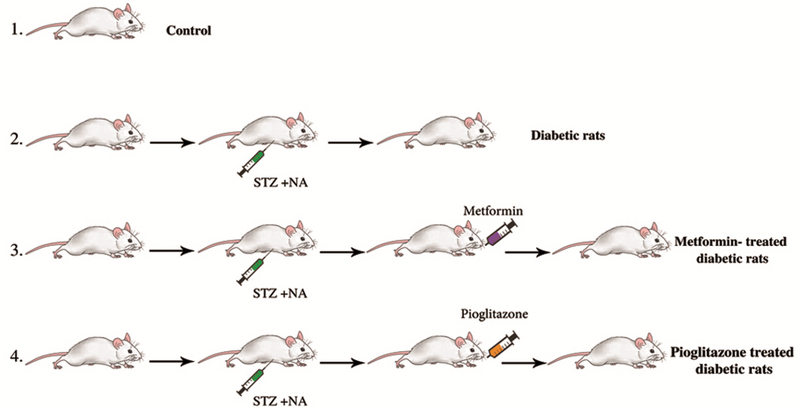
Animal grouping: I: control group; II: type 2 diabetic rats without any treatment; III: type 2 diabetic rats treated with Metformin; IV: type 2 diabetic rats treated with Pioglitazone. STZ, Streptozotocin; NA, Nicotinamide.

Twenty eight rats were randomly divided into four groups. The first group was the control group (Ι), including healthy rats without any intervention. The second group consisted of NA-STZ-induced type 2 diabetic rats without any treatment (IΙ) (FBS ≥ 250 mg/dl). The third group comprised of type 2 diabetic rats treated with 100 mg/kg/day Metformin (ΙΙΙ) (Sobhan, Iran) (17), and the fourth group was type 2 diabetic rats treated with 20 mg/kg/day Pioglitazone (IV) (Sobhan, Iran) (18) (Figure 1). Animals were kept in diabetic conditions for 4 wk (more than one sexual cycle). The hypoglycemic drugs were applied by orogastric gavage for another 4 wk. During all diabetic conditions and treatment procedures, FBS level was controlled by glucometer (HemoCue Glucose 201+, Sweden) and glucose reagent strips (ACCU-CHEK Active, Germany) every four days.

**Figure 2:**
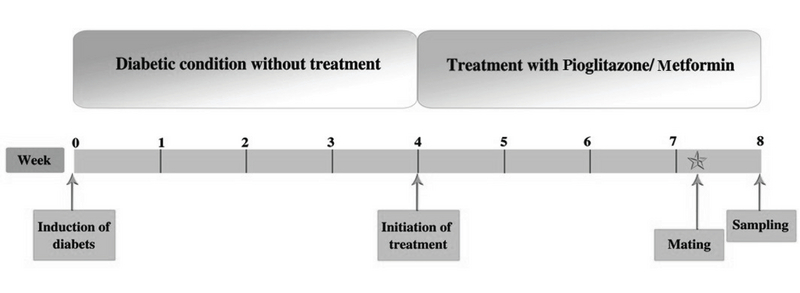
Study design. Animals were kept in diabetic conditions for 4 wk. Drugs were applied for another 4 wk. On the 52nd day, female rats were mated with male rat. On the third night, rats fasted overnight, were anesthetized on the following day, and then sacrificed in sterile conditions on the fourth day of pregnancy.

In the next step, on the 52nd day, two female rats of each group were mated with a male rat and vaginal plug was checked the following morning. The day on which the vaginal plugs were observed or vaginal smears showed spermatozoa was considered as the first day of pregnancy. On the third night, rats fasted overnight, were anesthetized by intraperitoneal injection of Ketamine Hydrochloride (ROTEXMEDICA, Germany) and Xylazine Hydrochloride (Daroupakhsh, Iran) on the following day (50 mg/kg and 7 mg/kg, respectively), thereafter, they were sacrificed in sterile conditions on the fourth day of pregnancy, the day of implantation (19) (Figure 2). Uterine horns were surgically removed, and all samples were snap-frozen in liquid nitrogen and stored at -80°C for further analysis.

### Total RNA isolation and cDNA synthesis

Total RNA was extracted from isolated ‎‏endometrial tissue using RNX-plus (Sinaclon, AryoGenBiopharma Complex. Iran) based on the manufacturer's protocol. RNA integrity was determined by 1% agarose gel electrophoresis. Total RNA concentration of each sample was estimated using a NanoDrop device (Nanolytik, Germany) at an optical density of 260 nm and stored at -80°C for the next step. DNase treatment was performed in order to omit genomic DNA in the RNA samples by DNase set (Fermentas, Lithuania). cDNA synthesis was performed using 1 µg of total RNA, as previously prepared, by means of PrimeScriptTM RT reagent Kit (TaKaRa, Kusatsu, Japan) as stated in the protocol.

### Quantitative real-time polymerase chain reaction

The relative expression levels of *VEGFA* and *LIF* genes were determined by quantitative real-time reverse transcription-polymerase chain reaction (RT-PCR) in comparison with β*-actin* as a reference gene. The primers were designed using GeneRunner software (version 4.0; Hastings Software. Inc. Hastings, US) and the specificity of each primer was tested by BLAST (http: //blast. ncbi.nlm.nih.gov/Blast.cgi). The list of the primers is shown in Table I.

Real-time PCR was carried out by Applied BiosystemsStepOnePlus${}^{\mathrm{TM}}$ instrument using RealQ Plus ×2 Master Mix, green (high ROX) (AMPLIQON, Denmark). Standard cycling protocol was applied to perform Real-time PCR. Amplification condition included: denaturation at 95°C for 10 min, denaturation at 95°C for 15 sec, annealing at the specific temperature for each gene (according to Table I) for 60 sec, followed by extension for 15 sec at 72°C for 40 cycles. To minimize experimental errors, all samples were tested in a triplicated manner. Gene expression assessment was performed using the 2-ΔΔ CT  method (20).

### Protein extraction and Western blot analysis

One hundred mg of uterus tissue was homogenized in 1 ml of cold RIPA buffer (Radioimmunoprecipitation assay buffer) (Cytomatingene, Iran) according to the manufacturer's protocol. The samples were incubated on ice for 30 min, followed by centrifugation at 27,000 g for 20 min. According to the manufacturer's instructions, the supernatant contains total proteins. Protein concentration quantification was performed using Bradford assay. Polyacrylamide gel electrophoresis was conducted using 10% SDS Polyacrylamide gel, and the samples were then moved to Nitrocellulose membranes (Bio-Rad, US). The membranes were blocked with 5% skimmed milk, blotted with rabbit anti-LIF (Cat. no. STJ29846, St John's Laboratory, United Kingdom) and rabbit anti-VEGFA (Cat no. ABS82, Merck, US) primary antibodies (dilution of 1:250), and incubated overnight at 4°C. Next, the membranes were washed three times for 15 min with PBS containing 0.05% Tween 20 and incubated with mouse anti-rabbit IgG - HRP secondary antibody (Cat. No. sc-2357, Santa Cruze, Almania) at a dilution of 1:1,000 at room temperature for 90 min. Clarity Western ECL Substrate Detection Reagent (Cat. no. Bio-Rad, 170-5060, US) was used to visualize the blots under SABZ biomedical chemiluminescent system (SABZ Biomedicals, Iran). The densitometry of bands was normalized to β*-actin* (Cat. no. ab8226; Abcam, US) using Image software, version 1.8 (21) (http://rsb.info.nih.gov/ij/).

**Table 1:** Real-time primer sequences


**Primers**	**Sequence**	**Melting temperature (°C)**	**Annealing temperature (°C)**	**Amplicon size (bp)**
***βactin*** **-Forward**	5'-GCCTTCCTTCCTGGGTATG-3'	63.4	
**** ***βactin*** **-Reverse**	5'-AGGAGCCAGGGCAGTAATC-3'	63	60	178
**LIF-Forward**	5'-GTCTTGGCCACAGGGATTG -3'	64.4			
**LIF-Reverse**	5'-CGTTGAGTTGAGCCAGTTGAC-3'	64.5	61.4	163
**VEGFA-Forward**	5'-ACCTCACCAAAGCCAGCAC-3'	57.7			
**VEGFA-Reverse**	5'-CTTGCAACGCGAGTCTGTG-3'	57.4	54.4	190
LIF: Leukemia inhibitory factor; VEGFA: Vascular endothelial growth factor-a; bp: Base pair				

### Ethical consideration

All animal experiments complied with the principles of the Declaration of Helsinki. All experimental procedures were approved by the Isfahan University of Medical Sciences Institutional Animal Ethical Committee (IR.MUI.REC.1396.3.366).

### Statistical analysis

All statistical analyses were performed using the SPSS software, version 20.0 (SPSS Inc., US). Kolmogorov-Smirnov test was used to test the normality of the data. Real-time PCR and Western blot were repeated at least three times. All values were presented as mean ± standard error of the mean (SEM). Analysis of Variance (ANOVA) with post-hoc multiple comparisons were performed to detect statistical significance which was considered as p < 0.05.

## 3. Results

### The effect of Metformin and Pioglitazone treatment on blood glucose levels

Four weeks following the induction of diabetes (after the fixation of diabetes in animals), drug treatment was commenced for the two groups of animals whose blood glucose levels were controlled once every four days. As described elsewhere, on day 0, the FBS levels of groups IΙ, IΙI, and ΙV before starting the drug treatment were significantly higher than those in the group Ι. Four days after the drug therapy, the FBS levels in both IΙI and ΙV groups showed a significant decrease compared to the group ΙΙ. This reduction continued such that on day 12, the FBS levels of IΙI and ΙV groups were not significantly different from those in the group Ι (22).

### VEGFA expression at the transcript and protein levels

**Figure 3:**
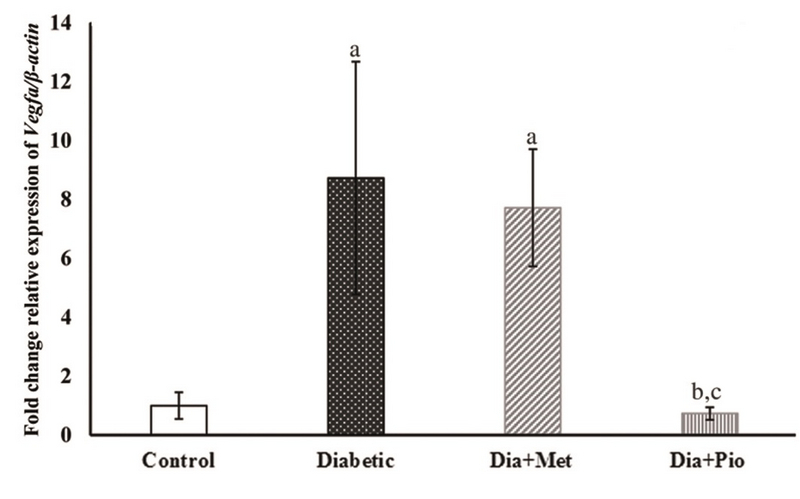
Relative expression of VEGFA transcript in rat endometrium at the time of implantation. The relative expression of *VEGFA* gene was normalized to β*-actin* using 2-ΔΔ CT  method. All values are presented as Mean ± SEM. P < 0.05 was considered statistically significant. Lowercase letters indicate the statistical significance as follows: compared to control (Ι) (a), untreated diabetic (ΙΙ) (b), and Metformin-treated diabetic (ΙΙΙ) (c) groups. Dia, Diabetic; Met, Metformin; Pio, Pioglitazone.

**Figure 4:**
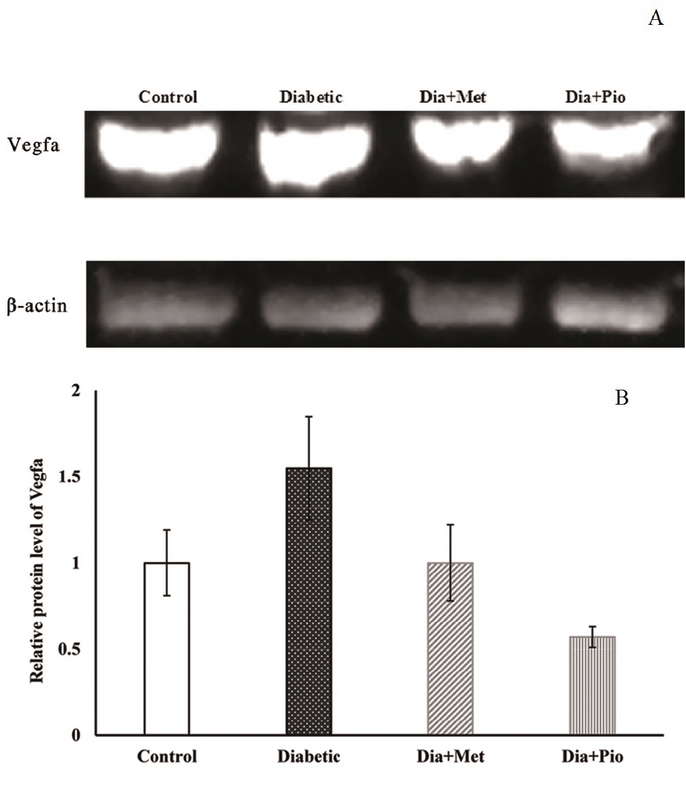
Relative expression of VEGFA protein in rat endometrium at the time of implantation. Western blot image of VEGFA and β-actin (as an internal control) proteins (A). The relative expression of VEGFA protein level was quantified by image J software (B). All values are presented as Mean ± SEM. P < 0.05 was considered statistically significant. Dia, Diabetic; Met, Metformin; Pio, Pioglitazone.

As shown in Figure 3, in comparison with group Ι, the relative expression level of VEGFA transcript was significantly higher in the diabetic (p = 0.02) and Metformin-treated (p = 0.04) (groups II and III) rats. Pioglitazone-treated (group IV) rats showed similar levels of VEGFA compared with group Ι. Furthermore, although the VEGFA transcript level was not statistically different between the untreated (group II) and the Metformin-treated (group III) diabetic rats, it was significantly reduced in the Pioglitazone-treated diabetic (group IV) rats (p = 0.03). The same trend was observed at the protein level of VEGFA although none of afore mentioned changes were statistically significant (Figure 4).

### LIF expression at the transcript and protein levels

**Figure 5:**
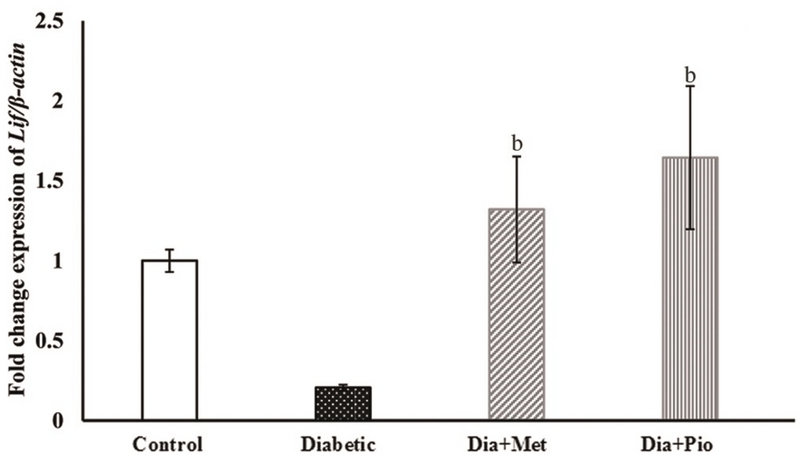
Relative expression of LIF transcript in rat endometrium at the time of implantation. The relative expression of *LIF* gene was normalized to β*-actin* using 2-ΔΔ CT  method. All values are presented as Mean ± SEM. P < 0.05 was considered statistically significant. Lowercase letters indicate the statistical significance as follows: compared to untreated diabetic group (II) (b). Dia, Diabetic; Met, Metformin; Pio, Pioglitazone.

**Figure 6:**
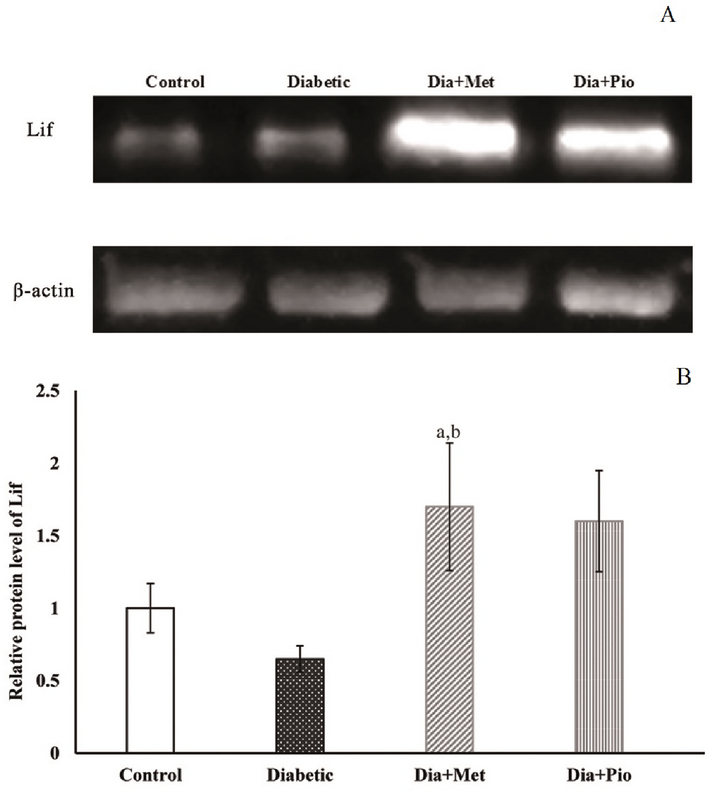
Relative expression of LIF protein in rat endometrium at the time of implantation. Western blot image of LIF and β-actin (as an internal control) proteins (A). The relative expression of LIF protein level was quantified by image J software (B). All values are presented as Mean ± SEM. P < 0.05 was considered statistically significant. Lowercase letters indicate the statistical significance as follows: compared to control (I) (a) and untreated diabetic (II) (b) groups. Dia, Diabetic; Met, Metformin; Pio, Pioglitazone.

The relative expression of LIF transcript decreased (though not significantly) in group ΙI compared to group Ι (p = 0.07). Moreover, compared to group Ι, the LIF expression was elevated (not statistically significant) in groups III and IV rats. Compared to group II, the expression of LIF transcript was significantly elevated in the ΙΙΙ (p = 0.01) and ΙV (p = 0.03) groups (Figure 5). The same trend was observed at the protein level of LIF although compared to group Ι, the Metformin-treated (group III) rats showed significantly higher levels of LIF protein (Figure 6).

## 4. Discussion

There is an association between the diabetic status and infertility in women, the main mechanism is yet to be fully fathomed. Moreover, the role of LIF and VEGFA in the implantation process and their aberrant expression in different tissues in diabetic condition has been reported. However, endometrial LIF and VEGFA expressions modulation during implantation in the context of diabetes is not well-understood. Accordingly, we aimed to investigate the endometrial expression changes of LIF and VEGFA at the time of implantation in diabetic rats following the treatment with Metformin and Pioglitazone.

VEGFA levels were observed to increase in the endometrial tissues in the diabetic conditions compared to group Ι. In accordance to our results, but in in vitro settings, Gu and colleagues observed that high glucose increased the percentage of VEGF${}^{+}$ uterus endometrial cancer cells, inducing VEGF secretion expression in cancerous cell lines (23). Similarly, Zeybek and colleagues showed that STZ-induced diabetic conditions significantly increased the Vegf expression in the uterine sections of diabetic rats, probably leading to abnormal angiogenesis (24). In contrast to the present study, their diabetic model was diabetes type 1, and they did not simulate the implantation conditions as their female rats did not mate with males.

In the present study, Metformin reduced (though not significantly) the diabetic-overexpression of VEGFA. In a similar manner, Yi and co-workers showed that the total expression of VEGFA was not reduced by Metformin treatment, following STZ-induced diabetic retinopathy (25). However, their diabetic model was type 1 diabetes, and they merely assessed VEGFA expression in mouse eyes. We further showed that Pioglitazone significantly reduced VEGFA expression compared to untreated diabetic rats. In the same manner and in a rat model of type 2 diabetic nephropathy, Xu and colleagues demonstrated that Pioglitazone can significantly reduce the hyperactivation of VEGFA in kidneys (26). To the best of our knowledge, there is no published report assessing endometrial VEGFA expression in the context of type 2 diabetes and treatment with Metformin and Pioglitazone during implantation.

Moreover, following the treatment with Metformin and Pioglitazone, we studied the endometrial expression changes of LIF at the time of implantation in a diabetic rat model. We observed that LIF expression was insignificantly reduced in the diabetic conditions. Similar to the present study, Albaghdadi and colleagues reported that LIF expression was downregulated in the uterine of nonobese diabetic mice in pre- and post-implantation processes (14). In contrast to the present findings and in a different tissue type, Broholm and co-workers observed that LIF expression was upregulated in T2DM condition in human muscle biopsy, although LIF downstream signals were impaired (9). However, our study is the first to investigate the LIF expression in the rat endometrium in the T2DM conditions.

In the current study, we further observed the upregulation of LIF following the treatment with Metformin and Pioglitazone in diabetic conditions. As far as we are concerned, no study has investigated the effect of Metformin and Pioglitazone on the endometrial expression of LIF in diabetic conditions.

Despite our novel findings, there existed certain limitations, such as the low number of rats in each group, possibly affecting the strength of the study. Moreover, the pregnancy of animals could be followed-up to investigate the molecular effects of drug treatments on pregnancy outcomes.

## 5. Conclusion

Endometrial expression of LIF and VEGFA is altered in diabetic rats during implantation which may be associated with diabetic-related infertility. It was further shown that Pioglitazone is able to more efficiently restore both VEGFA and LIF expressions to their baseline levels compared with Metformin. Further investigations are required to demonstrate the molecular mechanisms behind these gene expression alternations, which may play a key role in the interplay between diabetes and implantation failure.

##  Conflict of Interest

The authors declare that there is no conflict of interest.
